# Non-linearity of secondary pollutant formation estimated from emissions data and measured precursor-secondary pollutant relationships

**DOI:** 10.1038/s41612-022-00297-9

**Published:** 2022-09-14

**Authors:** Roy M. Harrison, David C. S. Beddows, Chengxu Tong, Seny Damayanti

**Affiliations:** 1 School of Geography Earth and Environmental Science, University of Birmingham, Edgbaston, Birmingham B15 2TT UK; 2 Department of Environmental Sciences, Faculty of Meteorology, Environment and Arid Land Agriculture, King Abdulaziz University, Jeddah, Saudi Arabia

**Keywords:** Environmental chemistry, Policy

## Abstract

In order to predict the impacts of reductions in air pollutant emissions, it is important to know whether secondary pollutant concentrations will decline in direct proportion to the reduction in their precursor, referred to as linearity. Trends in airborne concentrations of nitrate, sulfate, and SOC at sites in southern England are compared with emissions and concentration trends for sulfur dioxide (SO_2_), oxides of nitrogen (NO_*x*_), and non-methane VOC, and show some increased ratios of concentrations to emissions, strongly suggestive of non-linearity in the primary-secondary pollutant relationships for nitrate, but not the other pollutants. Analysis of a further 20-year dataset from the AGANET network shows a decline of nitrate concentrations significantly lower than that of NO_*x*_ emissions and ambient NO_*x*_ concentrations. For sulfate, the decline lies between that of emissions and airborne concentrations of SO_2_. Back trajectory analysis and Potential Source Contribution Function mapping for 2014–2018 show that the highest concentrations of secondary constituents in southern England are associated with air masses originating in mainland Europe, with 42% of sulfate, 55% of nitrate, and 35% of SOC estimated to be associated with air masses entering the UK from the European mainland.

## Introduction

A decline in the emissions and hence airborne concentrations of primary pollutants will in most cases lead to a reduction in concentrations of related secondary pollutants. However, the decline in secondary pollutant concentration may not be proportional to the reduction of its precursor, a phenomenon known as non-linearity. In general, non-linearity works in the sense of a less than proportionate decrease in the secondary pollutant, and hence it is likely that many secondary pollutant concentrations will not fall as rapidly as those of their main precursor.

Non-linearity is well known in gas phase atmospheric chemistry, for example in the oxides of nitrogen-ozone system. In polluted atmospheres, atmospheric photochemistry leads to net ozone production from photolysis of nitrogen dioxide (NO_2_) and peroxy radical chemistry^1^. This is most efficient at quite low concentrations of NO_2_, as NO_2_ is a sink for the hydroxyl radical (OH) which plays a key role in sustaining the photochemistry in which ozone is formed. Hence as NO_2_ concentrations reduce, at constant levels of Volatile Organic Compounds (VOC) and sunlight, ozone production efficiency rises leading to higher concentrations^2^. The increased OH radical concentration will cause enhanced oxidation of other compounds such as NO_2_, SO_2_, and VOC, leading potentially to more nitrate, sulfate, and secondary organic aerosol (SOA). This is likely to be a general phenomenon as many species such as VOC, SO_2_ and NO_*x*_ are sinks for atmospheric oxidants, and as they reduce in concentration, so oxidants may increase leading to faster formation rates of secondary pollutants. There are also cases of direct oxidant limitation, in which oxidant availability places a limit on the amount of a pollutant which can be oxidised, and a fall in precursor does not reduce the amount of secondary product if the precursor still exceeds the available oxidant^3^.

The secondary pollutants which contribute most to PM_2.5_ mass are typically sulfate, nitrate, and secondary organic compounds^4,5^. The clearest evidence of non-linearity deriving from atmospheric measurements is for sulfate. Emissions of SO_2_ in Europe and North America have reduced hugely in past decades, and this is reflected in lower measured concentrations of both SO_2_ and sulfate. Jones and Harrison^6^ examined the decline in concentrations at the EMEP sites across Europe, finding a far less than linear reduction in sulfate relative to SO_2_. Measurements in North America^7^ have similarly shown a less than linear reduction in sulfate. Evidence from measurements is much weaker for nitrate and secondary organic compounds, in the former case due to a much shorter history of emissions reductions for NO_*x*_ than for SO_2_, and in the latter case, the difficulty of making definitive measurements. However, reduced road traffic emissions during periods of lockdown due to Covid-19 have provided an opportunity for evaluation of precursor-secondary component relationships^8,9^, although these studies are more susceptible to effects of variations in weather than the longer-term datasets. Li et al.^10^ show complex non-linear responses of nitrate aerosol to controls on NO_*x*_ emissions in major cities of China, as did Balamurugan et al.^11^ in German cities. A recent paper by Pye et al.^12^ shows a high degree of linearity between emissions of SO_2_, NO_*x*_*,* and VOC and their ambient concentrations over the period 2002 to 2019 in the United States. The associated model study shows an interaction between pollutants, as well as regional variations in secondary pollutant reductions, which are mostly less than linear.

Numerical models are the only means of predicting future changes in secondary pollutant concentrations. These present particular difficulties and consequent uncertainties, which may be greater than for other pollutants. Specific issues include the ones described below.

Oxidation of SO_2_ to form sulfate takes place both in the gas phase by oxidants such as the OH radical and Criegee biradical, and in the liquid (cloud and aerosol) phase by ozone (O_3_) and hydrogen peroxide (H_2_O_2_) and by oxygen, catalysed by trace metals. Recent work in China has revealed a number of other mechanisms occurring at higher SO_2_ concentrations^13^, but it is currently unclear whether these are significant under European conditions. Many of the liquid phase processes are highly pH-dependent, and some are subject to limits on mass transfer into the aqueous phase. This level of complexity is beyond that which can be realistically incorporated into chemistry-transport models, and hence the processes are normally heavily simplified through parameterisations that are hard to test with measurement data.

The main daytime mechanism of nitrate formation is through reaction of NO_2_ with the OH radical to form nitric acid vapour. The nitric acid may react with marine sodium chloride, basic mineral compounds such as carbonates, and most often, predominantly with ammonia (NH_3_) to form particulate nitrates^14^. Reaction rates with the particulate components depend upon the size distribution and degree of hydration, and although rapid, the reaction with NH_3_ to form ammonium nitrate is reversible, and depends upon air temperature and humidity^15^. Due to the dependence upon NH_3_, nitrate concentrations will also be affected by NH_3_ emissions changes, and reductions in sulfate can lead to increase of nitrate due to enhanced NH_3_ availability^16^. Nitrate may also form at night-time through reaction of NO_2_ with ozone to form the NO_3_ radical which reacts with a further NO_2_ molecule to form N_2_O_5_ which is hydrolysed heterogeneously to nitrate^17^. Reaction rates are dependent upon several variables, including the rate of the vertical mixing of the atmosphere which is needed to maximise the contact of NO_2_-rich air from the surface with ozone-rich air from aloft to form NO_3_ radical^18^. Most models use highly simplified parameterisation of such processes to reduce computational expense, which may well be justified by the lack of definitive information upon the many complex environmental factors which determine conversion rates.

The oxidation of VOC gives rise to more oxygenated secondary molecules of lower vapour pressure which may condense into the particle phase to form SOA^19^. There are many VOCs that act as precursors of SOC, and their oxidation occurs by a wide range of both homogeneous and heterogeneous chemical processes^20^. The complexity of both VOC composition and oxidation pathways means that precursor-SOC relationships are very poorly quantified and are typically represented in chemistry-transport models by a few precursors and simple processes (e.g., Simpson et al.^21^). Consequently, predictions of the response of SOA to changes in VOC emissions have a high level of uncertainty.

As a consequence of the uncertain performance of numerical models with respect to the simulation of precursor-secondary pollutant relationships, there are benefits in learning from measurement datasets. These need to have run consistently for a period of several years in order for trends to be clearly detectable, and such datasets are available from the UK national networks and are analysed here. The aim is to evaluate the precursor-secondary pollutant relationships and identify any clear non-linearities.

## Results

### The urban sites

#### Trends in nitrate and nitrogen oxidation ratio (NOR)

**Fig. 1:**
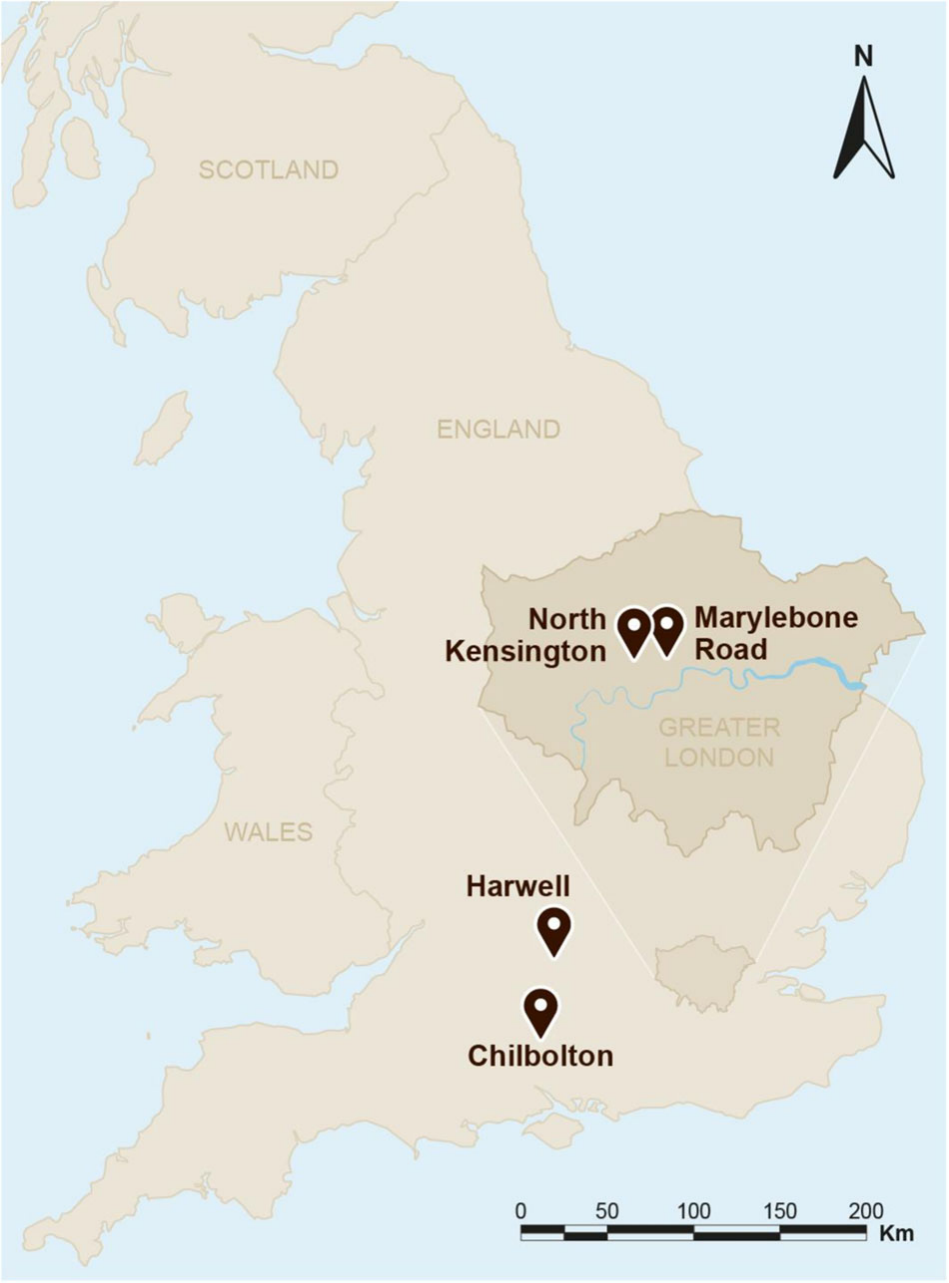
Map showing the site locations. (This map has been entirely created the authors of this paper).

Temporal trends in data collected at two sites in London were evaluated: the roadside site at Marylebone Road (LMR), and the central urban background site at North Kensington (LNK) (see Fig. 1).

**Fig. 2:**
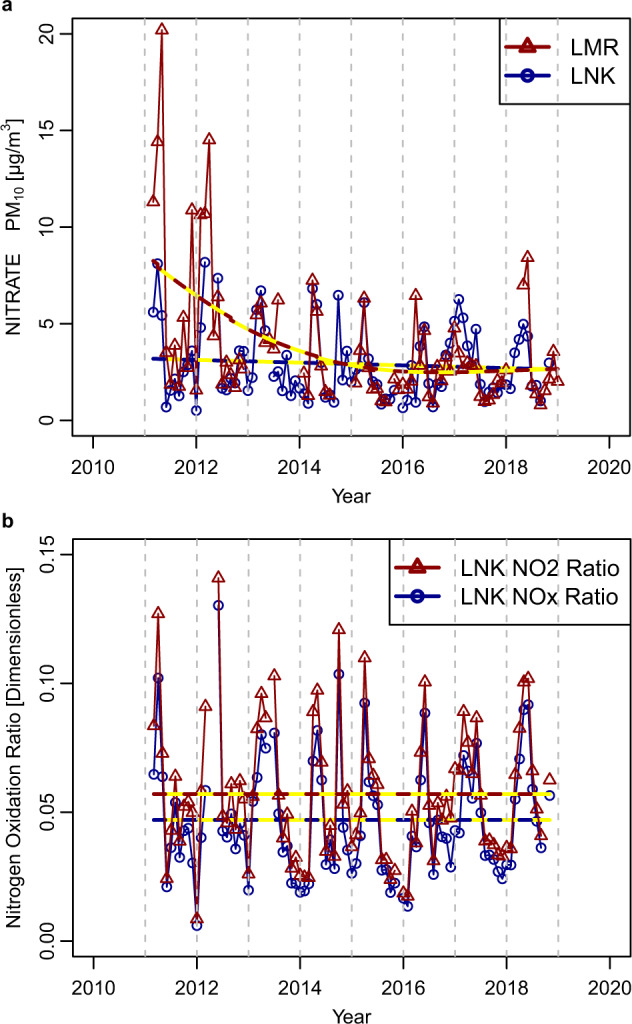
Trends in nitrate and nitrogen oxidation ratio at London sites. **a** Nitrate concentrations (monthly averages; µg m^−3^) at Marylebone Road (LMR) and North Kensington (LNK), and **b** the nitrogen oxidation ratio (dimensionless) with respect to NO_2_ and NO_*x*_, at LNK, 2011–2018.

Temporal trends in nitrate measured at LMR and LNK from 2012 to 2018 are given in Fig. 2, and the difference between the sites (LMR-LNK) in Supplementary Fig. 1. It is very clear that there has been no obvious difference between the sites since 2013, reflecting the nature of nitrate as a regional secondary pollutant. Concentrations of nitrate have changed little since 2014 according to the smoothed curve in Fig. 2. The diurnal, monthly and day-of-the-week variations of nitrate, NO_*x*_ and NO_2_ at the two sites are shown in Supplementary Fig. 2, and appear very similar—in particular for nitrate—at the two sites. The diurnal variation of nitrate is dominated by the reduced concentration in the afternoon due to evaporation as temperature increases and relative humidity falls^22^. Dilution in a deepening boundary layer will also contribute to a decline in the early afternoon. The monthly pattern is strongly influenced by the spring maximum associated with regional transport at a time when temperatures remain mostly quite low. This is a regular feature of nitrate in southern England, associated with frequent trajectories from the near continent at this time of year, and elevated NH_3_ emissions in upwind regions^23,24^. Surprisingly, there is a small decline in nitrate concentrations at weekends probably reflective of regional formation influenced by road traffic NO_*x*_ emissions.

The trends in the nitrogen oxidation ratio (NOR; defined as the ratio of nitrate to the sum of nitrate and NO_2_ or NO_*x*_) over the same period at LNK appear in Fig. 2. These are expected to be more meaningful for the background LNK site than roadside LMR which shows some unusual features in NO_*x*_ emissions^25^, and show very little change over the entire period, 2011–2018. Gradients determined by the Theil–Sen method are very small and not significantly upward or downward (Supplementary Table 1). Both the NOR and SOR (see below) are subject to influences of local sources of the precursor gases, especially in urban areas, and may be unreliable or unstable for this reason.

#### Trends in sulfate and the sulfur oxidation ratio (SOR)

**Fig. 3:**
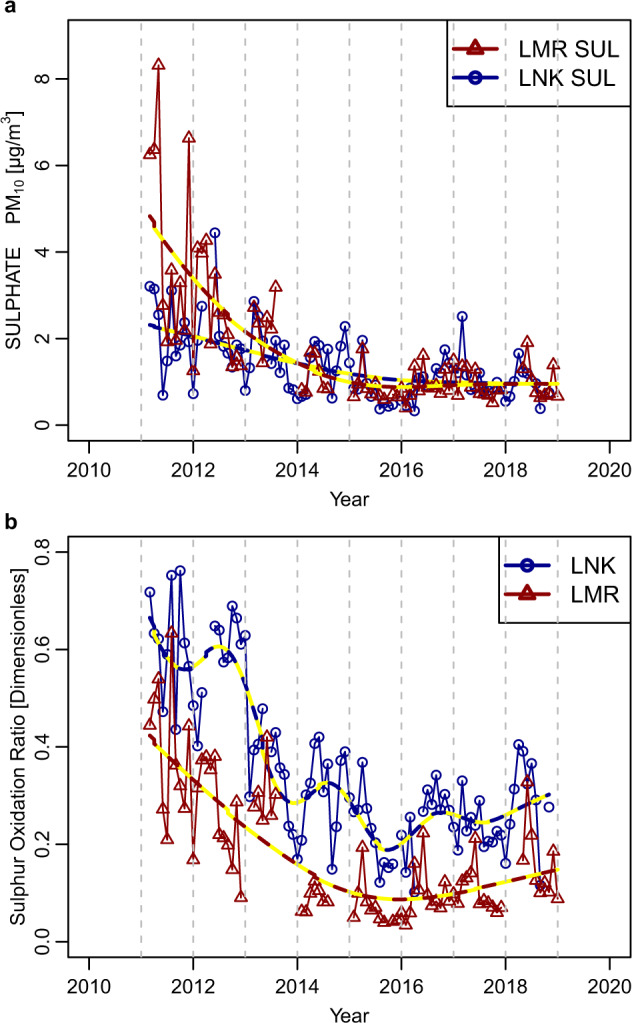
Trends in sulfate and sulfur oxidation ratio at London sites. **a** Sulfate concentrations (monthly average; µg m^−3^), and **b** sulfur oxidation ratio (dimensionless) at London, Marylebone Road (LMR), and London North Kensington (LNK), 2011–2018.

The trends in sulfate measured at LMR and LNK appear in Fig. 3. The concentrations show a decline, sharper at LMR than LNK, from 2011 to 2014. After that date, the two curves become almost coincident, with no marked difference between the sites, again indicative of a regional secondary pollutant, and very little change in concentrations.

The diurnal, monthly, and day-of-the-week variations in sulfate (Supplementary Fig. 3) show much similarity to nitrate, but with a smaller decline in the afternoon, presumably related to a deepening boundary layer, rather than evaporative loss. Much the same monthly pattern is seen as for nitrate, suggesting a strong influence of long-range transport. The reduced concentrations at weekends are perhaps more surprising given the generally slower oxidation of SO_2_ than NO_2_ to form secondary aerosol.

The Sulfur Oxidation Ratio (SOR; ratio of sulfate to the sum of SO_2_ and sulfate) at both sites shows a reduction from 2011 to 2014, to reach a minimum, and the curves then increase steadily to the end of the dataset in 2019 (Fig. 3). A notable caveat is that SO_2_ concentrations are close to the detection limit, which introduces uncertainty into the SOR.

#### Trends in elemental (EC) and organic carbon (OC)

Temporal trends in elemental carbon (EC) appear in Supplementary Fig. 4 for both sites. There has been a rapid decline at LMR corresponding to the introduction of diesel particle filters which became mandatory on new vehicles with the introduction of the Euro 5 standard in 2011. The decline at LNK is much less rapid, but nonetheless steady over this period (Supplementary Fig. 4 and Supplementary Table 1).

The trends in OC are in the same direction, but to a lesser extent (Supplementary Fig. 5). This much slower decline is because of the large contribution of SOA at both sites which has probably changed little with time, and the fact that diesel particle filters are more effective for removing EC than OC, as much of the latter is semi-volatile and can penetrate the filter as vapour and then condense into the particulate phase in the cooler environment of the atmosphere.

The EC/OC data were studied in more detail by calculating an approximate secondary organic carbon (SOC) fraction by three methods.

Method 1 assumes that all primary OC and all EC derives from road traffic and hence:

$${{{\mathrm{SOC}}}} = {{{\mathrm{OC}}}}_{{{{\mathrm{total}}}}}-{{{\mathrm{OC}}}}_{{{{\mathrm{traffic}}}}}$$

For this purpose, OC_traffic_ for the LNK site was calculated from:

$${{{\mathrm{OC}}}}_{{{{\mathrm{nk}}}}\;{{{\mathrm{traffic}}}}} = {{{\mathrm{EC}}}}_{{{{\mathrm{nk}}}}}{{{\mathrm{x}}}}\left( {{{{\mathrm{OC}}}}_{{{{\mathrm{mr}}}}}-{{{\mathrm{OC}}}}_{{{{\mathrm{nk}}}}}} \right)/\left( {{{{\mathrm{EC}}}}_{{{{\mathrm{mr}}}}}-{{{\mathrm{EC}}}}_{{{{\mathrm{nk}}}}}} \right)$$

Method 2 was to use the EC tracer method^26,27^ which estimates SOC from:

$${{{\mathrm{SOC}}}} = {{{\mathrm{OC}}}}_{{{{\mathrm{total}}}}}-\left( {{{{\mathrm{OC/EC}}}}} \right)_{{{{\mathrm{min}}}}}{{{\mathrm{x}}}}\;{{{\mathrm{EC}}}}$$

where (OC/EC)_min_ is the lowest consistent measured ratio, which corresponds to periods of negligible SOC, and hence represents primary emissions.

Method 3 was the Minimum R Squared (MRS) method proposed by Wu and Yu^28^, which depends upon initial calculation of hypothetical SOC values based upon a range of (OC/EC)_primary_ values. The SOC concentration with the minimum correlation with EC is then selected as the most probable value. Wu and Yu^28^ regard this method as more conceptually sound than the EC tracer method, but it has not been widely adopted to date.

**Fig. 4:**
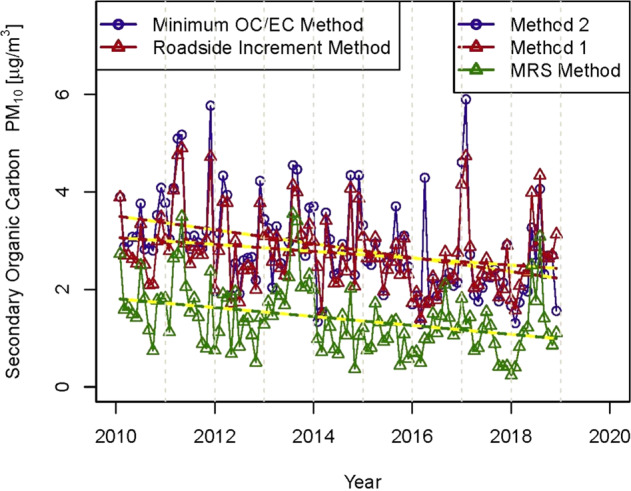
Trend in secondary organic carbon. Monthly averages (µg m^−3^) at North Kensington, 2010–2018, estimated by the binary method (Method 1), the EC tracer method (Method 2), and the Minimum R Squared (MRS) method (Method 3).

The first two methods gave the close predictions seen in Fig. 4. This is a little surprising as the EC tracer method has a tendency to underestimate SOC because it depends upon there being negligible SOC in the atmosphere at certain times, which may not be the case, and some SOC is then included in the primary component. On the other hand, the binary mixture method (Method 1)—which assumes that any OC which is not a primary emission from road traffic is SOC—is liable to over-estimate SOC if there are any other primary emission sources present. However, the MRS method estimates lower SOC than either of Methods 1 and 2, which is consistent with Method 2 overestimating SOC by failing to account for non-traffic primary OC, but hard to explain in relation to the result from the EC tracer method. In Fig. 4, all three methods show an appreciable overall decline from 2010 to 2018, but very little decline over the final three years of 2016 to 2018. The gradient over the entire period is significantly downward (Supplementary Table 1).

### The rural sites

These are part of the same measurement network as the London sites, and comprise two sites which operated sequentially. The Harwell site closed at the end of 2014 to be replaced by the Chilbolton site (see Fig. 1). Unfortunately, these sites are not as similar as had been hoped, and there are distinct discontinuities in the primary pollutant data, but less so for the secondary pollutants which are more subject to regional as opposed to local influences.

**Fig. 5:**
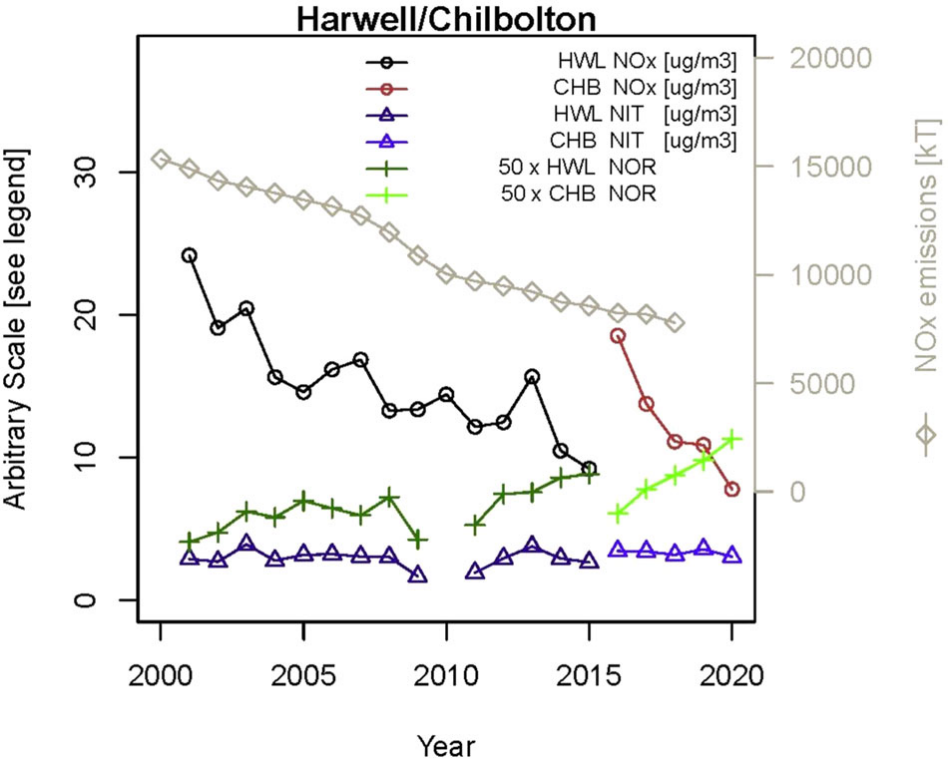
Trends in nitrogen metrics at Harwell and Chilbolton. Emissions of NO_*x*_, airborne concentrations of NO_*x*_ and nitrate, and nitrogen oxidation ratio at Harwell and Chilbolton, 2000–2020.

Figure 5 shows the trend in nitrate and its precursors, as well as NOR and the emissions summed across the United Kingdom, Belgium, Netherlands, Germany, Denmark and France. The latter (i.e., the summed emissions) is included because the primary pollutant concentrations are subject to influences of local sources, as is seen in the Harwell/Chilbolton combined dataset, while the emissions are more representative of the overall contribution of a precursor across the entire source area. Downward trends in emissions and concentrations of NO_*x*_ do not appear to be well reflected in reductions in nitrate, and the NOR is seen to increase in the latter period of the dataset.

Supplementary Fig. S6 shows sulfate and SO_2_ at Harwell/Chilbolton with a rising trend in SOR seen in the later years. In Supplementary Fig. 7, the emissions relate to non-methane hydrocarbons which appear to show a decline similar to that of Organic Carbon (OC) or SOC, although the discontinuity between the HWL and CHB site data makes a clear comparison of trends difficult.

### Quantification of relative trends

The temporal trends in secondary pollutants have been plotted as a ratio to emissions, with the earliest point set to 1.0 to allow trends to be viewed. These are shown for London, North Kensington in Supplementary Figs. 8–10, and for London, Marylebone Road in Supplementary Figs. 11–13, and for Harwell/Chilbolton in Supplementary Figs. 14–16. Considering the plots for North Kensington, there is no compelling evidence of non-linearity. Ratios after the first year tend to be >1.0, but do not rise steadily as might be expected with a strong non-linearity, and the NO_*x*_ and nitrate measurements do not diverge appreciably from one another. There is little indication of divergence from the emission trend for SO_2_ or sulfate. Organic Carbon shows a very flat trend with time relative to Non-methane volatile organic compound (NMVOC) emissions, and if the first year (2008) is discounted, the trend in SOC is very flat, and almost parallel to the NMVOC emissions trend. The Theil–Sen estimation in Supplementary Table 1 indicates significant upward trends in sulfate and nitrate relative to precursor emissions, but a downward trend in SOC.

The data for London, Marylebone Road (Supplementary Fig. 11) appear to show an increase for NO_*x*_ and nitrate relative to the emissions trend. The former may be linked to errors in emissions estimates for NO_x_ in the EEA data. The work of Krecl et al.^25^ shows a difference in the behaviour of NO_*x*_ at Marylebone Road relative to sites in Stockholm and Copenhagen, and higher emission factors than are used in many inventories. Hence, the trends in emissions taken from the European Environment Agency may be incorrect for all countries. In the case of SO_2_ and sulfate (Supplementary Fig. 12), there is little divergence of sulfate from the emissions trend, despite noise in the data, but a large divergence of SO_2_ in the latter part of the plot is suggestive of a local SO_2_ source. Both OC and SOC run parallel to the NMVOC emissions trend (Supplementary Fig. 13). None of the trends is significant (Supplementary Table S1), with wide confidence intervals due to the scatter. The Harwell/Chilbolton data suffer from the discontinuity caused by the move of the site, but show possible increased secondary/emissions ratios for nitrate (Supplementary Fig. 14), but not for sulfate (Supplementary Fig. 15), or SOC which shows some signs of a divergence downwards from the OC data and emissions trend (Supplementary Fig. 16). If such a trend is real, and not an artefact of the estimation method for SOC, it would suggest that NMVOC emissions reductions have been greatest for those compounds which are SOC precursors.

**Fig. 6:**
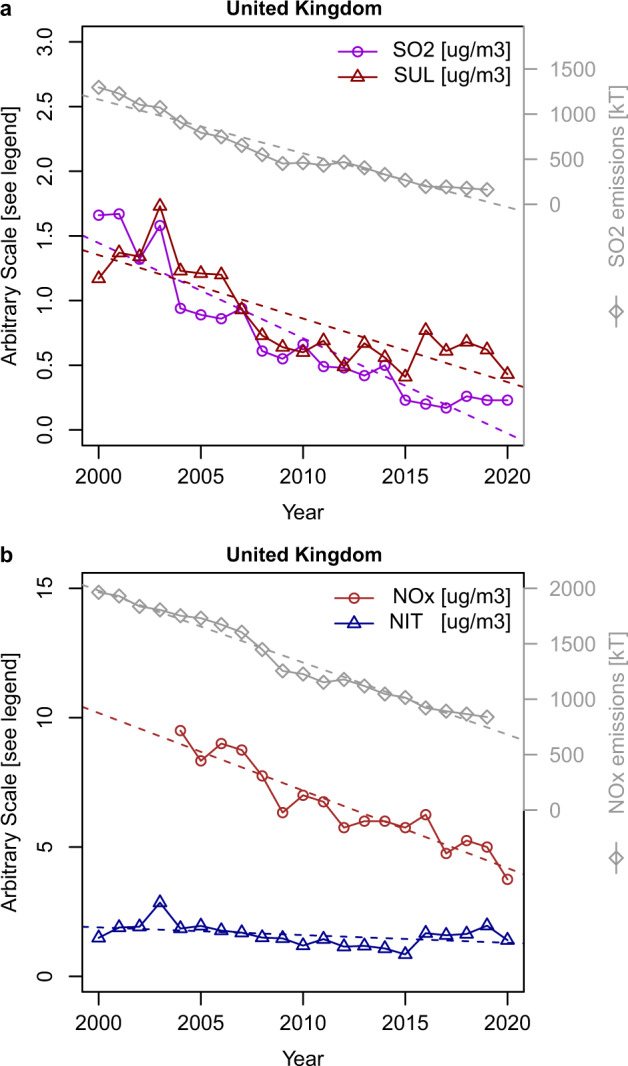
Trends in nitrate and sulfate from the AGANET sites. **a** UK emissions and concentrations of SO_2_, and sulfate concentrations, and **b** emissions and concentrations of NO_*x*_, and nitrate concentrations, from 2000 to 2020 from the AGANET data.

An additional view on rural trends has been derived from the UK Acid Gas and Aerosols Network (AGANET; https://uk-air.defra.gov.uk/networks/network-info?view=aganet) which is also a UK government-sponsored network that uses low flow rate DELTA samplers to capture gases and particles by samplers of gaseous pollutants and particulate matter, and has operated since 1999. There are now around 30 sites, but only 11 have operated continuously from 2000 to 2020. Tang et al.^29^ analysed trends in both primary and secondary components from this network between 2000–2015 (12 sites) and 2006–2015 (30 sites) together with primary pollutant emissions trends. They examined declines in nitrate and sulfate concentrations relative to the emissions of primary pollutants. The trends in SO_2_ and sulfate, and in NO_*x*_ and nitrate in our analysis are shown in Fig. 6. In this case, emissions data from the UK alone are used as the vast majority of AGANET sites are in rural and remote areas of Scotland and Northern Ireland, and receive much less influence from mainland European sources than the London and southern England sites analysed above. Trend lines have been fitted using the Theil–Sen method in Openair. The same European composite emissions dataset (as used above) was also plotted, and the trends are very similar to the UK dataset.

In the case of SO_2_, only the emissions data give a really close fit to a linear trend (Fig. 6) and there is some difficulty in fitting the early and late years in the SO_2_ and sulfate datasets. However, at 5.59% (95% confidence interval, 5.43–5.97%) per year, the decline in SO_2_ gas is slightly larger than the emissions trend (4.49%, 95% CI: 4.49–4.50% per year). The downward trend in sulfate at 4.36% (95% CI: 4.01–4.70%) is slower than that of SO_2_ and very similar to that of the SO_2_ emissions, for which the confidence intervals overlap. The AGANET network does not measure NO_*x*_, so data were taken for four collocated rural sites which are operated as part of the Enhanced Urban and Rural Network. The trends, shown in Fig. 6 show a very close fit to a downward emissions trend of 2.84% (95% CI: 2.81–2.86%) per year, with NO_*x*_ concentrations at the four sites falling slightly faster (3.48%, 95% CI: 3.48–3.51%). On the other hand, nitrate concentrations at the 20 sites fell at 2.12% (95% CI: 1.54–2.66%), significantly slower than both NO_*x*_, and the emissions trend. Not plotted in Fig. 6 is the trend in Σ(particulate nitrate plus nitric acid), which is 2.35% (95% CI: 1.88–2.82%) per year which, like particulate nitrate will be affected by NH_3_ emissions and nitricacid-NH_3_ reactions, and is similar to the trend in particulate nitrate. Tang et al.^29^ evaluated the percentage relative median change in concentrations of nitrate and sulfate over the periods 2000–2015 and 2006–2015 and compared it with the trends in emissions and concentrations of gas phase precursors. They did not include confidence intervals, but generally found very similar trends in precursor emissions and concentrations, and secondary aerosol components. Tang et al^29^. reported declines in sulfate (−5.4% per annum) slightly lower than the emissions trend for SO_2_ (−6.5% per annum) over the period 2006–2015, and that trends in nitrate and NO_*x*_ emissions were not significantly different. Their analysis provides clear evidence of non-linearity for sulfate, but not nitrate.

### Trajectory cluster analysis

**Fig. 7:**
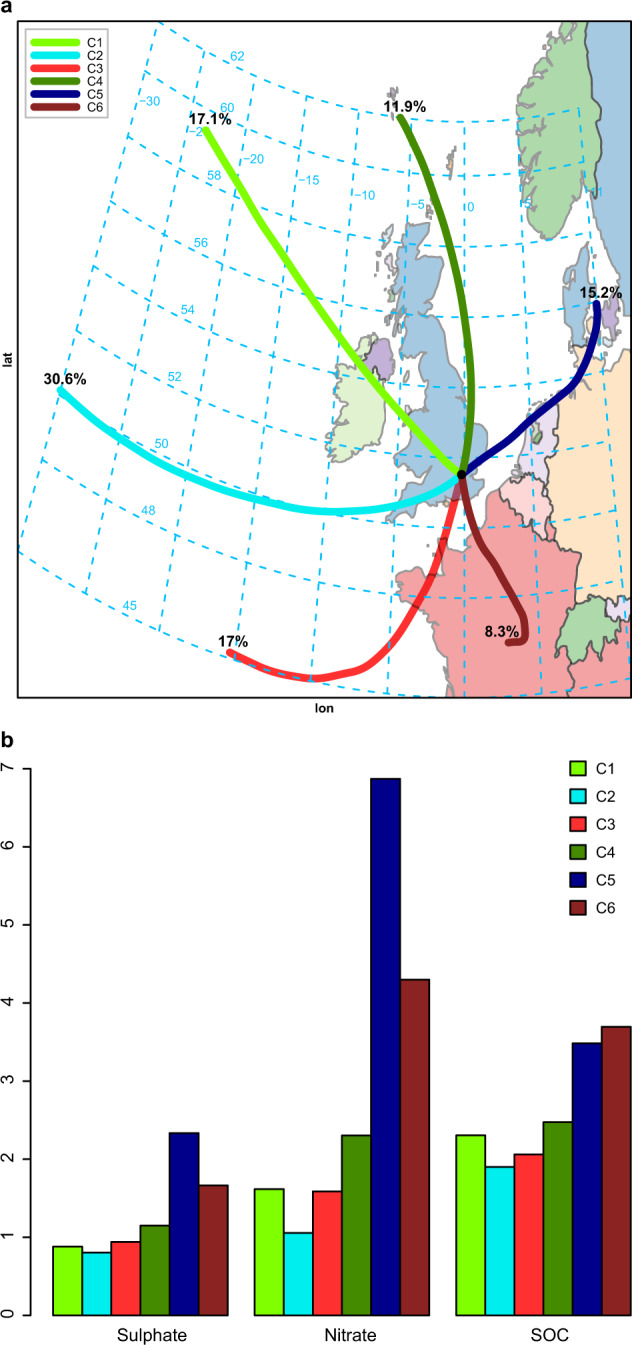
Results of air mass clustering. **a** Clustered air mass back trajectories for air masses arriving at the LNK site, and **b** mean concentration of nitrate, sulfate, and SOC associated with each back trajectory. Cluster 1: polar maritime, Cluster 2: returning polar maritime, Cluster 3: tropical maritime, Cluster 4: arctic maritime, Cluster 5: polar continental, and Cluster 6: tropical continental.

Air mass back trajectories (3-day) were calculated and clustered for the years from 2014 to 2018 in a single analysis using Hysplit^30^. The results of the clustering appear in Fig. 7, and show the six airmass types which most commonly affect the UK^31^, i.e., polar maritime (Cluster 1), returning polar maritime (2), tropical maritime (3), arctic maritime (4), polar continental (5) and tropical continental (6).

Average concentrations of nitrate and sulfate, and of the NOR and SOR associated with each trajectory appear in Table 1, in which numbers marked in green represent the lowest value within a year, and those in red are the highest value, and also in Fig. 7 for the secondary components. The highest average values for all secondary constituents apart from SOC and for the Oxidation Ratios are associated with Cluster 5 (polar continental), while the lowest averages for all secondary constituents, without exception, and Oxidation Ratios are associated with Cluster 2 (returning polar maritime). The highest concentrations of primary pollutants (and SOC by a small margin) are associated with the slow-moving (short) trajectories of Cluster 6 (tropical continental) from the south, usually associated with rather stagnant weather conditions.

**Table 1:** Average concentration of each chemical species and oxidation ratio associated with each of the back trajectory clusters shown in Fig. 7.

Cluster	SO_2_	Sulfate	S.ratios	NO_2_	NO_*x*_	Nitrate	Ratios.NO_2_	Ratios.NO_*x*_	EC	OC	SOC	OC/EC
C1	2.09	0.88	0.21	24.6	40.5	1.62	0.045	0.033	0.96	3.23	2.31	*3.85*
C2	2.06	*0.80*	*0.21*	*23.4*	*35.1*	*1.06*	*0.034*	*0.025*	*0.67*	*2.43*	*1.90*	4.22
C3	*2.03*	0.94	0.23	24.8	36.3	1.59	0.043	0.033	0.73	2.69	2.06	4.38
C4	2.39	1.15	0.28	26.6	42.4	2.30	0.057	0.041	0.85	3.24	2.47	4.38
C5	2.54	**2.33**	**0.36**	33.8	59.4	**6.87**	**0.118**	**0.085**	0.99	4.48	3.48	**5.11**
C6	**2.67**	1.66	0.29	**38.3**	**76.2**	4.30	0.081	0.058	**1.32**	**4.99**	**3.70**	4.22

The bold font indicates the trajectory with the highest mean concentration for the species, and the italicised font the lowest.

### Origins of high concentrations and mass fluxes

**Fig. 8:**
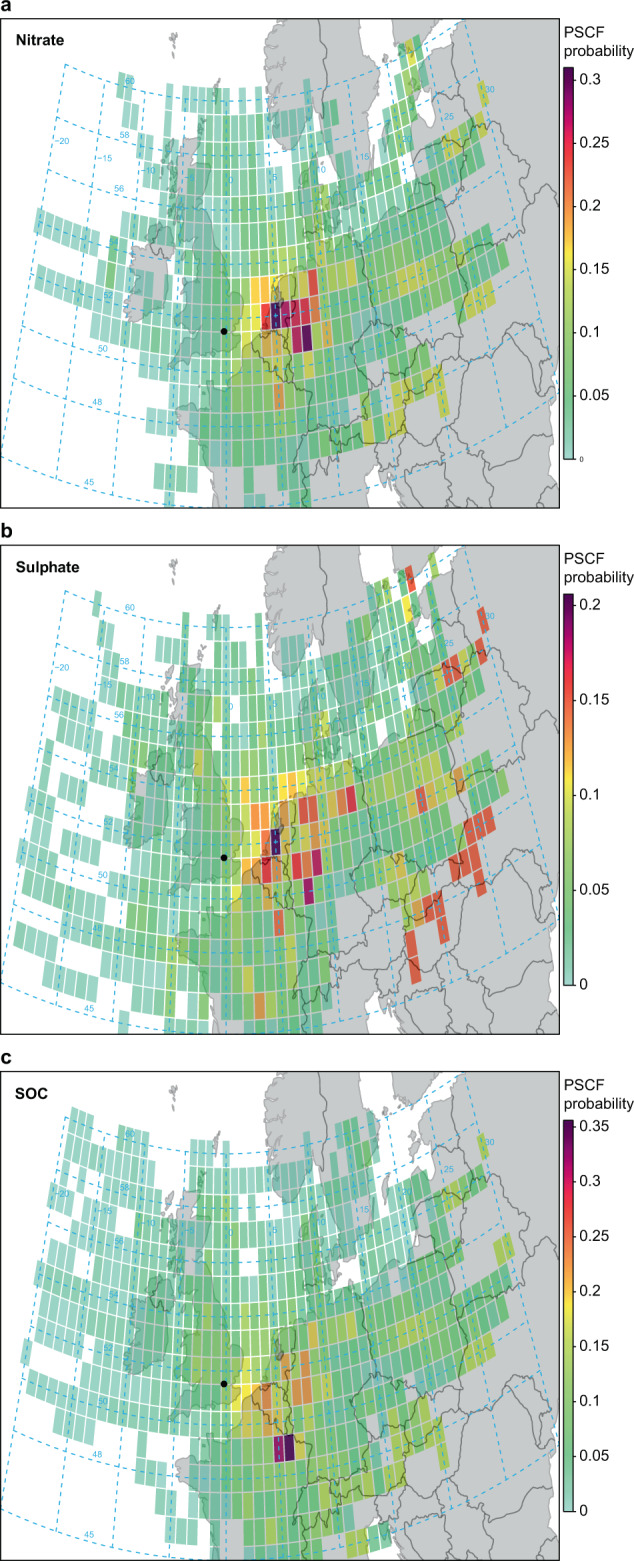
Potential source contribution analysis for trajectories from 2014 to 2018. The specified constituents are **a** nitrate, **b** sulfate, and **c** secondary organic carbon.

The trajectory analysis was used in greater depth to evaluate the origins of high concentrations using a function in Openair. The results of a Potential Source Contribution Function analysis appear in Fig. 8. This shows that high pollutant concentrations are more prevalent when the trajectories originate from the east, which is seen from the high values in the plot. The main source areas for high concentrations of nitrate are in western mainland Europe, including Belgium, the Netherlands, Denmark, Germany and France. For sulfate, there is also a larger contribution from eastern Europe, probably reflecting the higher density of SO_2_ emissions and slower oxidation of SO_2_ than NO_*x*_. The sources of high concentrations of SOC are less well defined, but with a markedly more southerly emphasis than for the inorganic species, possibly reflecting a larger contribution of biogenic emissions to SOC in the warmer, more southerly air masses.

The contributions of different trajectories to mass fluxes of pollutants are shown in Table 2. This shows the result of calculating the product of trajectory frequency (*f*_*i*_) and concentration (*C*_*i*_) and normalising by the total for the six trajectories, i.e.,

$${{{\mathrm{Mass}}}}\;{{{\mathrm{flux}}}}\left( {{{{\mathrm{trajectory}}\,i}}} \right) = {{{f}}}_i.{{{C}}}_i/\Sigma {{{f}}}_i.{{{C}}}_i$$

**Table 2:** Percentage mass flux of each chemical constituent associated with each back trajectory cluster.

Cluster	SO_2_	Sulfate	NO_2_	NO_*x*_	Nitrate	EC	OC	SOC
C1	16%	13%	16%	16%	11%	19%	17%	16%
C2	**28%**	21%	**27%**	**24%**	13%	**24%**	**23%**	**24%**
C3	16%	14%	16%	14%	11%	15%	14%	14%
C4	13%	*12%*	*12%*	*11%*	11%	*12%*	*12%*	*12%*
C5	17%	**30%**	19%	20%	**41%**	18%	21%	22%
C6	*10%*	12%	12%	14%	14%	13%	13%	13%

The bold font indicates the trajectory with the highest mean concentration for the species, and the italicised font the lowest.

This shows the highest mass fluxes of sulfate and nitrate to be associated with the continental trajectory, Cluster 5. In combination, the two continental trajectories which contain largely imported pollution account for 42% of sulfate, 55% of nitrate, and 35% of SOC, which serves to emphasise the importance of mitigation of sources in mainland Europe as well as the UK, if secondary particle concentrations are to be substantially reduced. These estimates should be considered approximate as each clustered trajectory contains a wide range of individual trajectories, hence blurring the UK/mainland Europe divide, and concentrations in the continental trajectories will contain a component derived from oxidation of both continental and UK emissions over the UK land mass. A further complicating factor is the large magnitude of shipping emissions in waters around the UK, which will contribute to all of the trajectories, but not equally.

## Discussion

The close similarity of both nitrate and sulfate concentrations between the roadside Marylebone Road and urban background North Kensington sites are typical of a regional pollutant with no immediate local sources. The back trajectory analysis shows clearly that the source regions responsible for the highest concentrations of nitrate, sulfate, and SOC in southern England are in mainland Europe. The other countries appearing to have greatest responsibility (Belgium, Netherlands, Germany, Denmark, France), together with the United Kingdom were used to create emissions trends for SO_2_, NO_*x*_, and NMVOC.

The relationships between precursors and secondary products have been examined in two ways. Firstly, the temporal trends in precursor and secondary pollutant concentrations are examined. These show some evidence of increasing secondary to precursor ratios. The NORs show a mixed picture with no obvious trend at London, North Kensington (LNK), but a distinct upward trend in the data for both rural sites, Harwell and Chilbolton. There is more consistency in SORs, which are increasing at all urban and rural sites. Concentrations of SOC are decreasing in the measurement data.

The weakness in the NOR/SOR method is that the concentrations of both product and precursor are measured in the same location, hence introducing an assumption the trends in the precursor at the source site are the same as those at the receptor site. Consequently, the relationships of pollutant concentrations to emissions estimates were evaluated. These again show a rather mixed picture, with the strongest evidence for non-linearity forthcoming for nitrate from almost all sites, with the data for sulfate being more equivocal. Interpretation of the data for SOC is more complex, as it contains a component formed from biogenic VOC which are not included in the emissions inventory data for NMVOC, and hence measured SOC are not expected to decline as fast as anthropogenic NMVOC emissions, even if that component shows linearity in concentrations. Nonetheless, there is no obvious divergence in trends in measured OC or estimated SOC from the NMVOC emission trend. However, there is good evidence in the dataset from London and southern England, consistent with other studies and numerical models, of non-linearity in the secondary pollutant-precursor relationships for nitrogen, but not for sulfur which will make concentration reductions for the secondary constituents and hence also for PM_2.5_ concentrations especially difficult to achieve. A second analysis using the 11-site AGANET dataset also shows a less than linear decline in nitrate, but not in sulfate, when judged against both emissions and precursor (NO_x_ and SO_2_) concentration trends. Hence, there is a consistency between the datasets.

Analysis of air mass back trajectories shows both the highest concentrations and highest mass fluxes for the secondary pollutants are associated with continental trajectories, highlighting the need for action across Europe to mitigate secondary aerosol pollution in the UK.

## Methods

### Sources of data and methods of analysis

Data were taken from the UK national air quality archive (https://uk-air.defra.gov.uk/data/). Nitrate and sulfate are measured as part of the Particle Size and Composition Network using URG 9000B analysers with a PM_10_ inlet. A Partisol 2025 sequential air sampler with Ultrapure quartz filters (Pallflex Tissuquartz) is used for the sampling of PM_10_ Elemental Carbon and Organic Carbon (EC/OC). Analysis of the filters is conducted with a Sunset Laboratory OC/EC analyser following the EUSAAR 2 protocol^32^. Full details of the instruments, procedures, and quality assurance are given in National Physical Laboratory^33^.

Data are taken from two sites in a central area of London. London, Marylebone Road (LMR) is located in a street canyon on a six-lane highway with 80,000–90,000 vehicles per day. An analysis of the many air pollution datasets collected at this site is presented by Kamara and Harrison^34^. The other site, in the central urban background, is London, North Kensington (LNK), located at about 4 km from the LMR site. An analysis of earlier data from this site is presented by Bigi and Harrison^35^. The difference in concentrations between these sites has been taken as representing the increment due to road traffic above the urban background in a number of studies (e.g., Harrison and Beddows^36^). Data were also taken from two rural sites in southern England. The Harwell site operated until 2015, after which it was replaced by Chilbolton. Details of all sites are available from https://uk-air.defra.gov.uk/networks/search-site-info, except the closed Harwell site, which is described in Charron et al.^37^. Figure 1 shows the site locations.

In addition to time series of concentrations, the trends are also examined in the following:

$${{{\mathrm{Nitrogen}}}}\;{{{\mathrm{Oxidation}}}}\;{{{\mathrm{Ratio}}}}\left( {{{{\mathrm{NOR}}}}} \right){{{\mathrm{ = Nitrate - N/}}}}({{{\mathrm{Nitrate - N + NO}}}}_2\left( {{{{\mathrm{or}}}}\;{{{\mathrm{NO}}}}_{{{x}}}} \right){{{\mathrm{ - N}}}})$$

$${{{\mathrm{Sulphur}}}}\;{{{\mathrm{Oxidation}}}}\;{{{\mathrm{Ratio}}}}\left( {{{{\mathrm{SOR}}}}} \right){{{\mathrm{ = Sulphate - S/}}}}({{{\mathrm{Sulphate - S + SO}}}}_2{{{\mathrm{ - S}}}})$$

Pollutant emissions data for SO_2_, NO_*x*_, and non-methane VOC were downloaded from the National Emission reductions Commitments (NEC) Directive emission inventory data website, (https://www.eea.europa.eu/data-and-maps/data/national-emission-ceilings-nec-directive-inventory-18), and are the annual sum of emissions from Belgium, Netherlands, Germany, Denmark, France, and the United Kingdom for the years 1990–2018 for comparison with the southern England sites described above.

Data for nitrate, sulfate and SO were also downloaded from UK-Air for the AGANET sites. AGANET is operated on behalf of Defra by the UK Centre for Ecology and Hydrology. The following 11 sites were used: Bush Estate, Cwmystwyth, Eskdalemuir, Glensaugh, High Muffles, Lough Navar, Rothamsted, Stoke Ferry, Strathvaich, Sutton Bonnington, and Yarner Wood. Site locations can be viewed on: https://uk-air.defra.gov.uk/interactive-map?network=aganet and http://www.pollutantdeposition.ceh.ac.uk/aganet, and the sampling and analytical systems are described in http://www.pollutantdeposition.ceh.ac.uk/ammonia_methodology. Full site and method details have also been reported by Tang et al.^29^, who report trends in data from 1999 to 2015. One notable feature of the DELTA sampler used in AGANET is that the inlet has a 50% cut point at around 4.5 µm diameter, so the smaller end of the coarse fraction is sampled, along with the fine fraction in a PM_4.5_ fraction. Also notable is the fact that the sampler incorporates a denuder and therefore samples acid gases as well as particles. Data for nitric acid vapour are used in this work, alongside condensed phase data for nitrate and sulfate. Due to the distribution of sampling sites right across the UK, emissions data for the UK alone were used with this dataset.

## Supplementary information

Supplementary Material

## Data Availability

Data supporting this publication are openly available from the UBIRA eData repository at 10.25500/edata.bham.00000763.

## Code availability

No codes were used to support this study.
