# Supplementary material for: Non-linearity of secondary pollutant formation estimated from emissions data and measured precursor-secondary pollutant relationships
Source: NPJ Clim Atmos Sci. 2022 Sep 14;5(1):71. doi: 10.1038/s41612-022-00297-9 (PMC9471023; doi:10.1038/s41612-022-00297-9)
Supplement: Supplementary file 1 — Supplementary Material [file 41612_2022_297_MOESM1_ESM.pdf]

Supplementary Information

Non-Linearity of Secondary Pollutant Formation Estimated from Emissions Data and Measured Precursor-Secondary Pollutant Relationships at UK Sites

Roy M. Harrison*, David C. S. Beddows, Chengxu Tong and Seny Damayanti

*** Corresponding author: Roy M. Harrison (r.m.harrison@bham.ac.uk)**

Contents of this file

Supplementary Table 1

Supplementary Figures 1 to 16

Supplementary Table 1. Percentage change values (△%) with their lower and upper 95% confidence limits (CL) calculated for each figure presented. Please refer to the y-axis of each figure for the exact details of each measurement referenced below.

Figure	Site	Measurement	Percentage change per year			Regression
			△%	*lower CL*	*upper CL*	
3	LNK	NO_2 Ratio	-0.39	-4.19	5.34	Theil-Sen
3	LNK	NO_x Ratio	0.22	-3.90	6.47	Theil-Sen
6	SOC	Method 01	-2.38	-3.95	-0.79	Theil-Sen
6	SOC	Method 02	-4.32	-5.33	-3.32	Theil-Sen
6	SOC	MRS Method	-5.20	-7.42	-3.08	Theil-Sen
S4	LMR	EC	-7.75	-7.91	-7.18	Theil-Sen
S4	LNK	EC	-4.17	-5.57	-2.34	Theil-Sen
S8	LNK	NO_x	2.29	0.92	3.65	Linear
S8	LNK	Nitrate	2.35	0.77	3.94	Linear
S9	LNK	SO_2	0.18	-1.69	2.06	Linear
S9	LNK	Sulphate	3.07	0.53	5.62	Linear
S10	LNK	OC	1.22	-0.47	2.90	Linear
S10	LNK	SOC	-3.82	-7.17	-0.47	Linear
S11	LMR	NO_x	4.90	3.65	6.15	Linear
S11	LMR	Nitrate	2.78	-3.30	8.86	Linear
S12	LMR	Sulphate	-1.20	-3.84	1.43	Linear
S12	LMR	SO_2	9.40	5.97	12.8	Linear
S13	LMR	OC	0.22	-2.19	2.63	Linear
S13	LMR	SOC	-0.93	-4.22	2.37	Linear

[LNK – London North Kensington; LMR – London Marylebone Road; MRS - Minimum R squared]

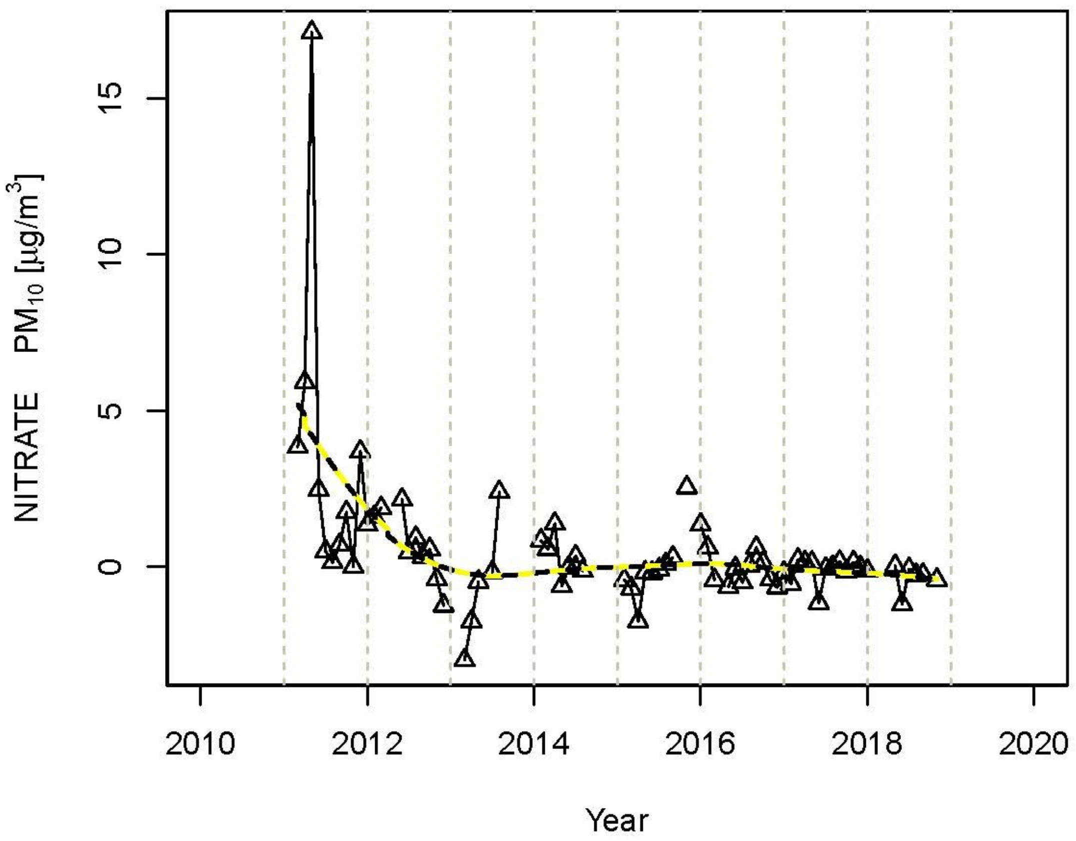

Supplementary Figure 1. Difference in nitrate concentration between sites (LMR – LNK), 2011 – 2019.

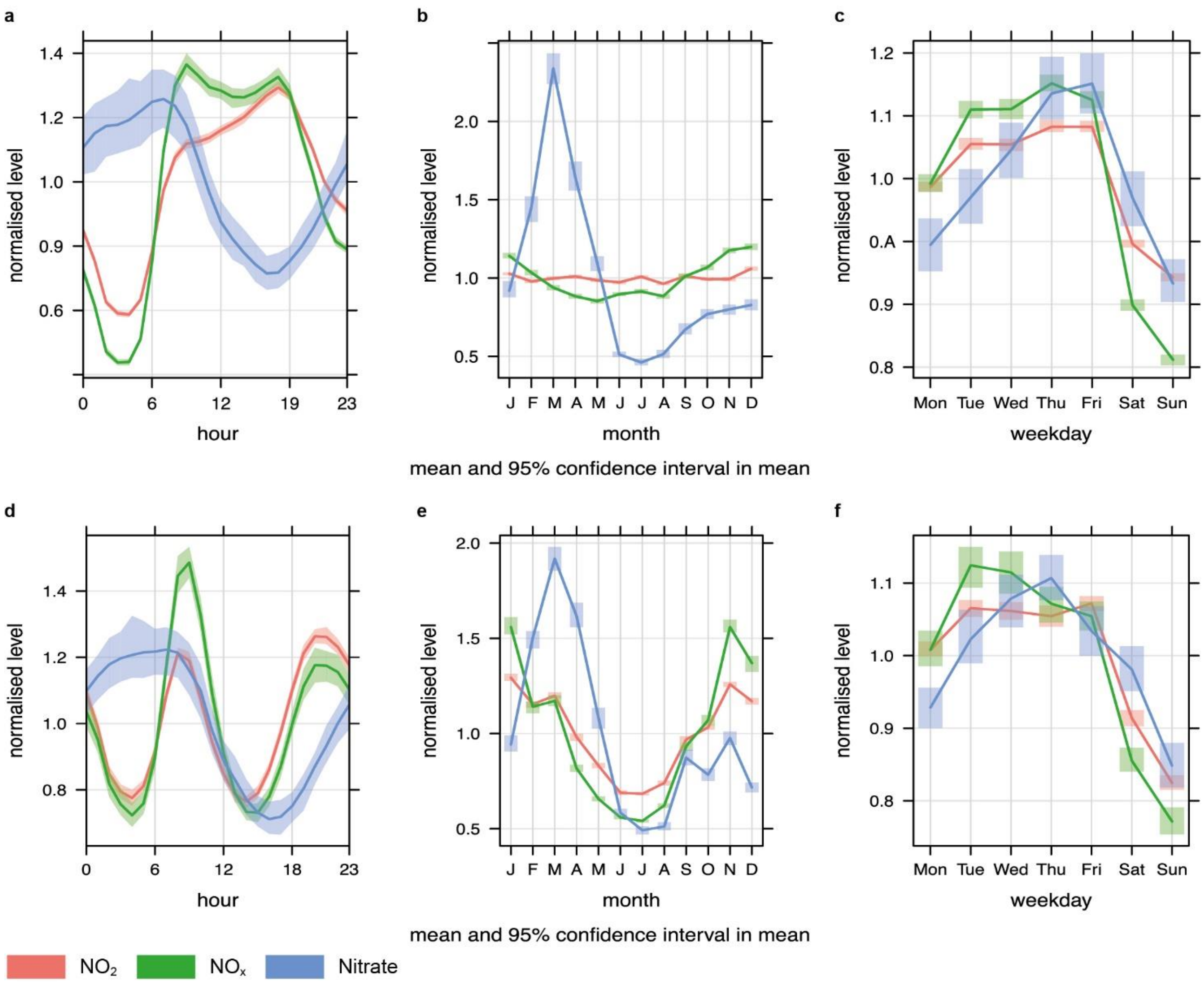

Supplementary Figure 2. Average diurnal, monthly and day-of-week trends in NO_x, NO_2 and nitrate at LMR (top) and LNK (bottom).

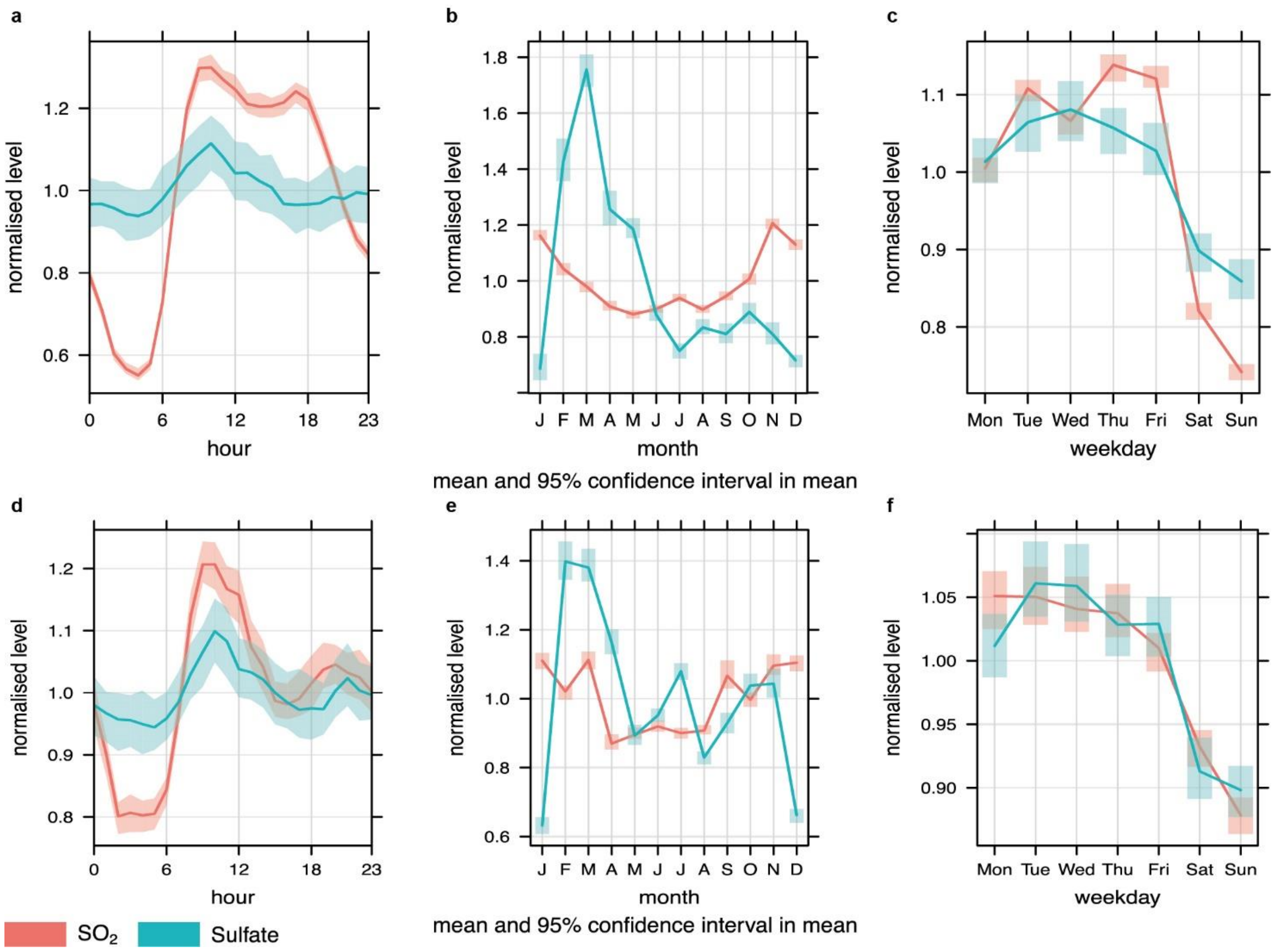

Supplementary Figure 3. Average diurnal, monthly and day-of-week variation in SO_2 (red line) and sulphate (green line) at LMR (top) and LNK (bottom).

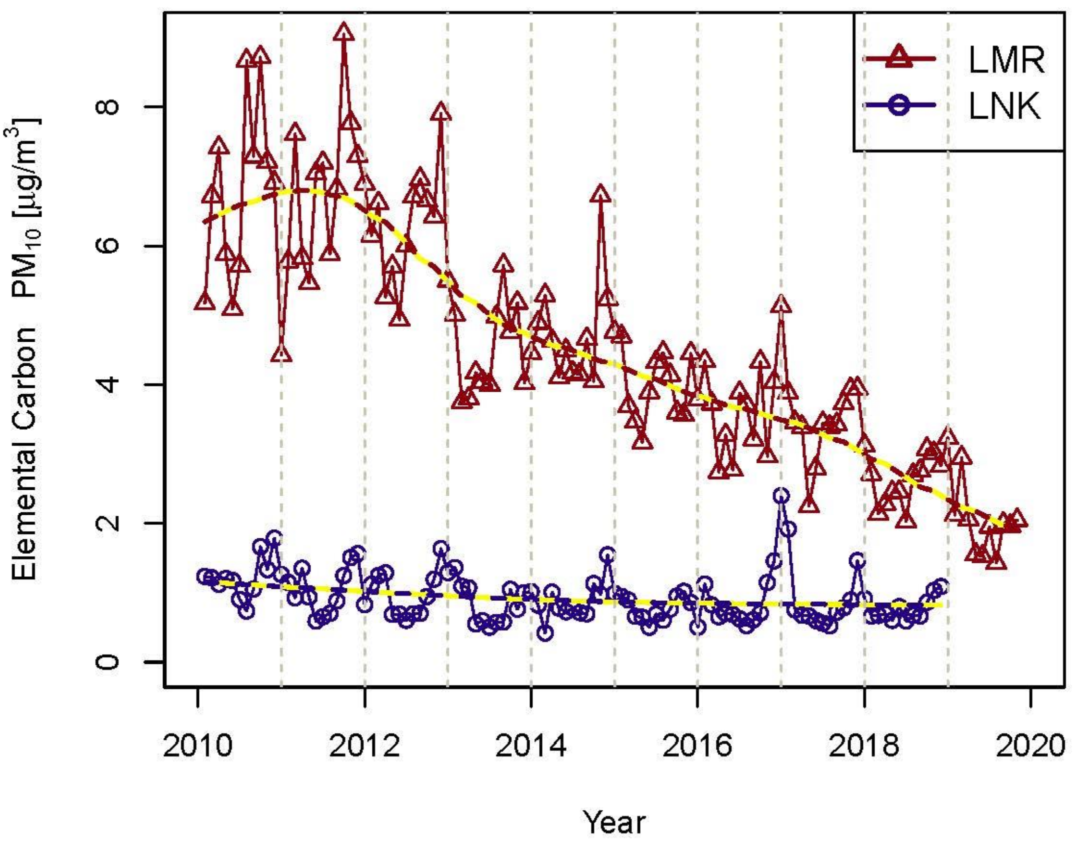

Supplementary Figure 4. Trends in Elemental Carbon (monthly averages) at LMR and LNK, 2010 – 2019.

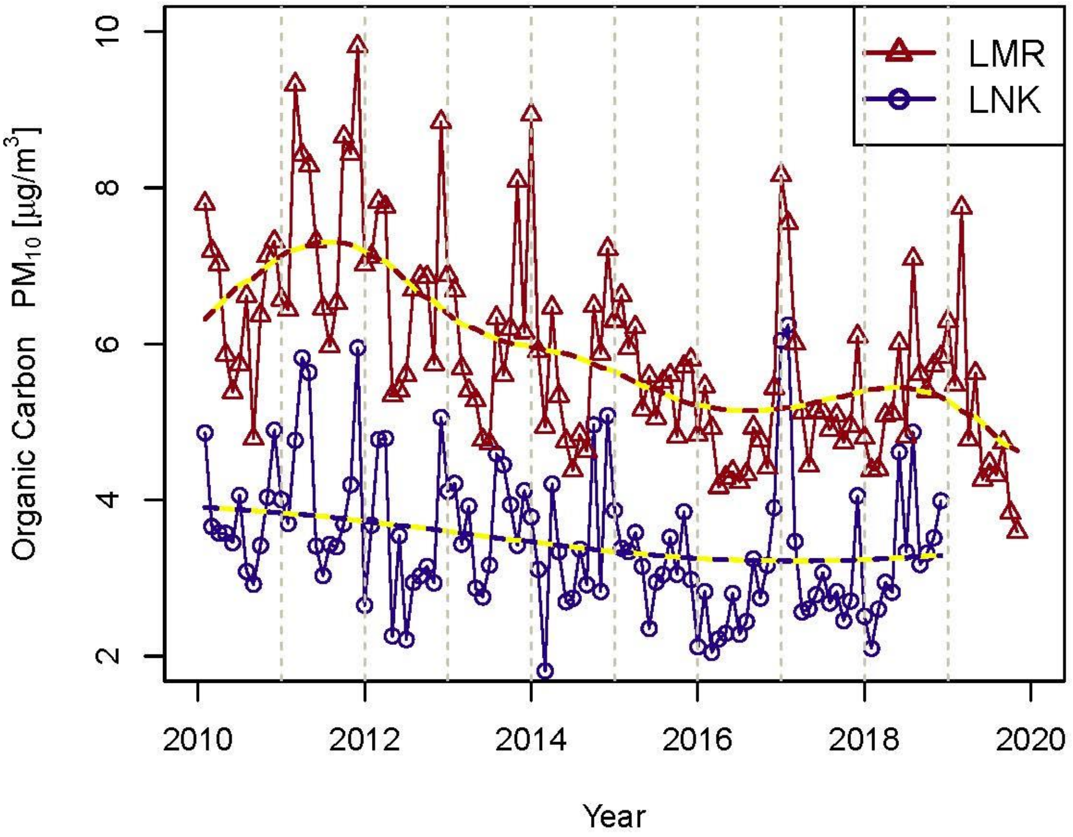

Supplementary Figure 5. Trends in Organic Carbon (monthly averages) at LMR and LNK, 2010 – 2019.

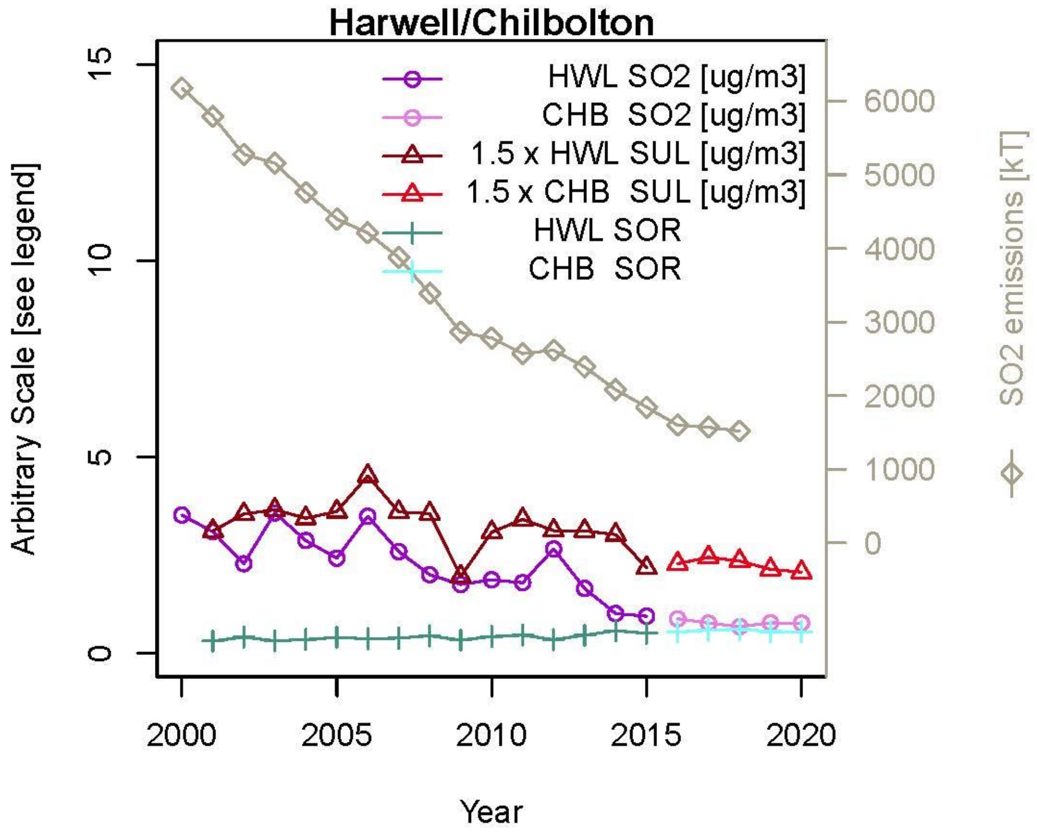

47 **Supplementary Figure 6. Trends in emissions of SO_2, airborne concentrations of SO_2**
48 **and sulphate, and sulphur oxidation ratio at Harwell and Chilbolton, 2000 – 2020.**

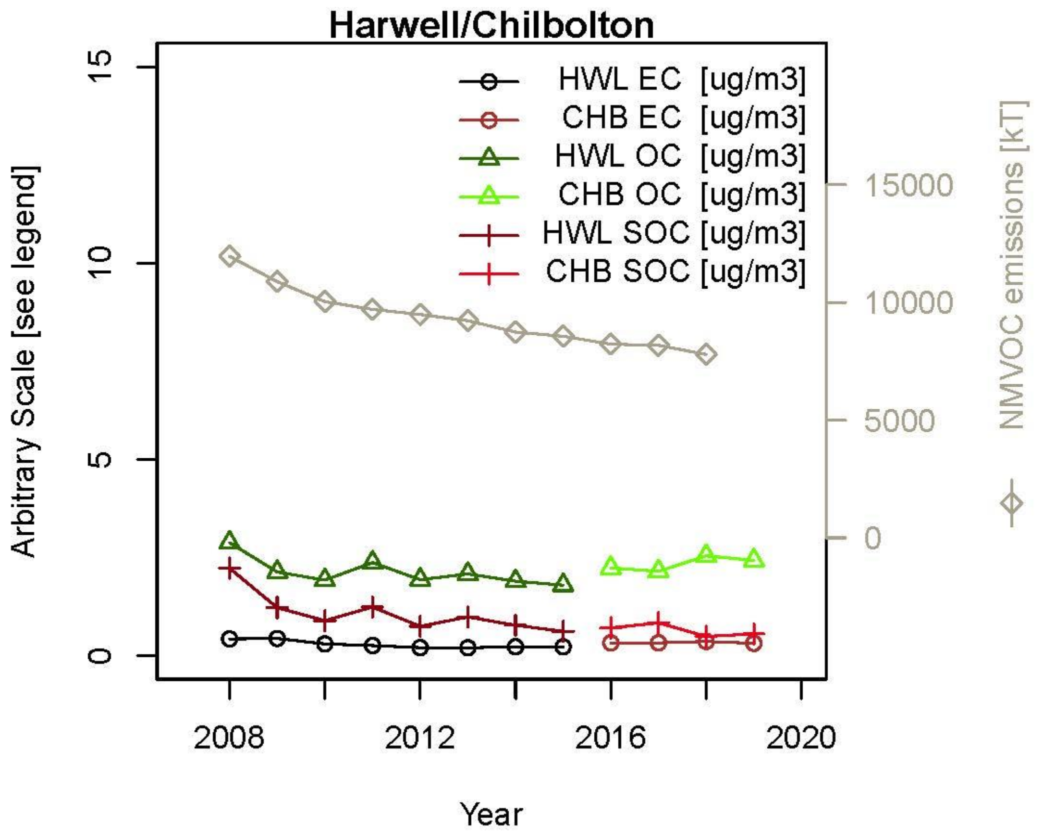

Supplementary Figure 7. Trends in emissions of NMVOC, and airborne concentrations of EC, OC and SOC at Harwell and Chilbolton, 2008 – 2019.

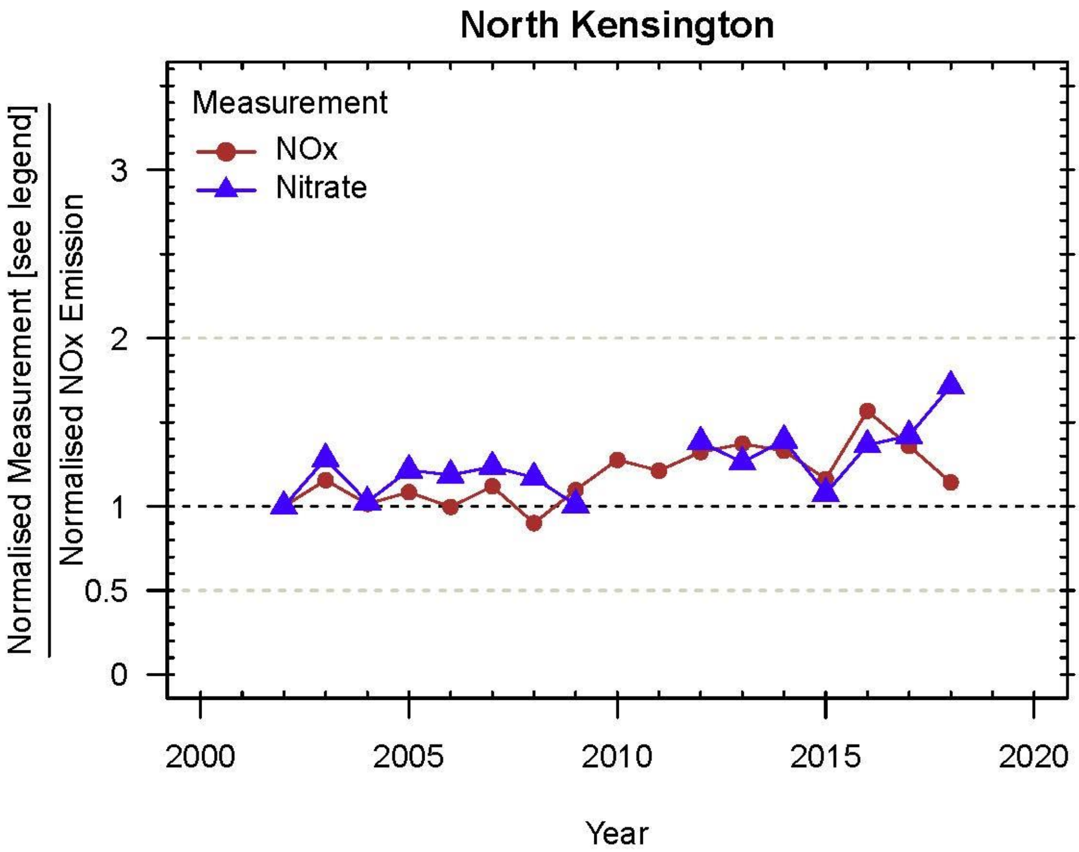

Supplementary Figure 8. Normalised ratios of concentrations of nitrate and NO_x to emissions of NO_x at North Kensington, 2002 – 2018.

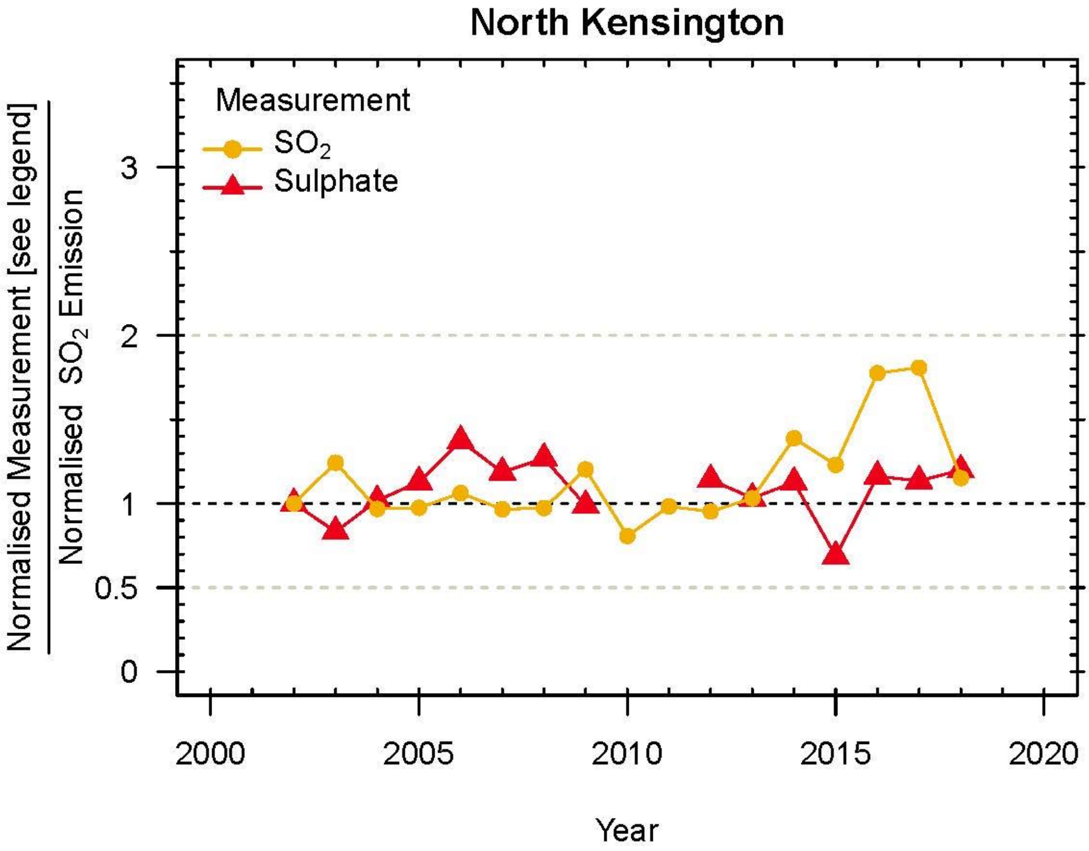

Supplementary Figure 9. Normalised ratios of concentrations of sulphate and SO_2 to emissions of SO_2 at North Kensington, 2002 – 2018.

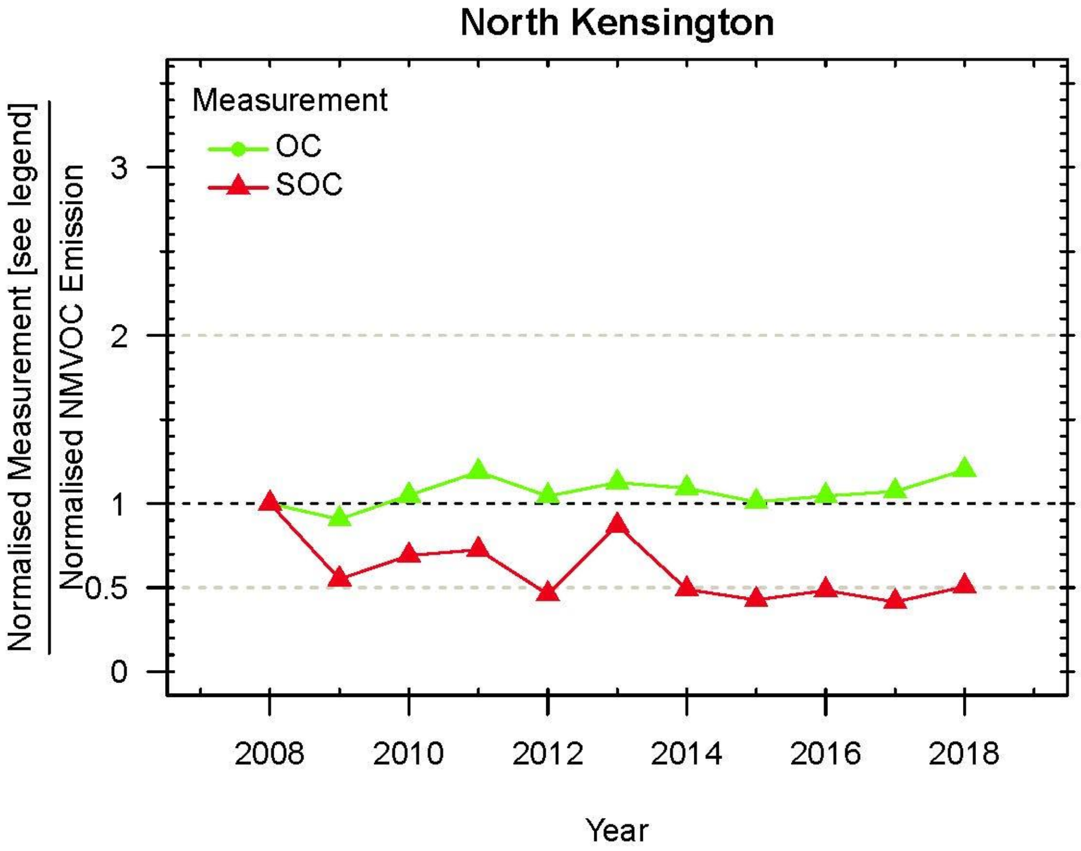

Supplementary Figure 10. Normalised ratios of concentrations of SOC and OC to emissions of NMHC at North Kensington, 2008 – 2018.

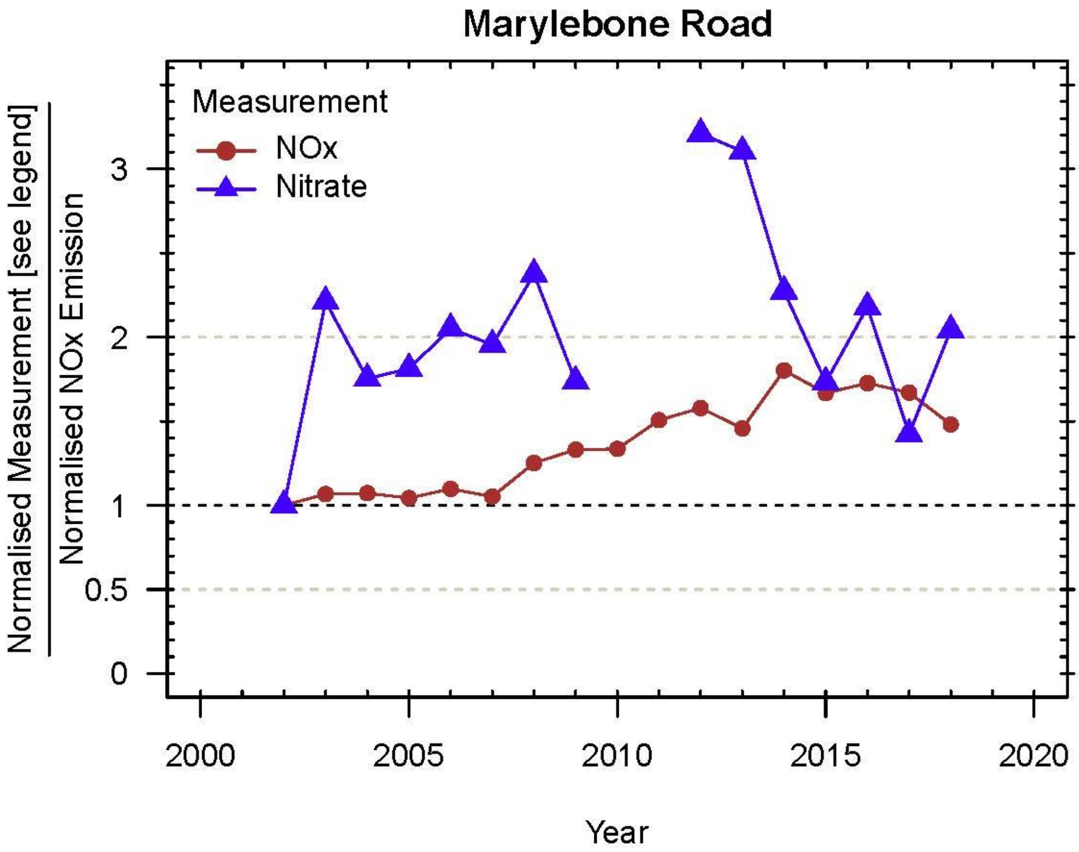

Supplementary Figure 11. Normalised ratios of concentrations of nitrate and NO_x to emissions of NO_x at Marylebone Road, 2002 – 2018.

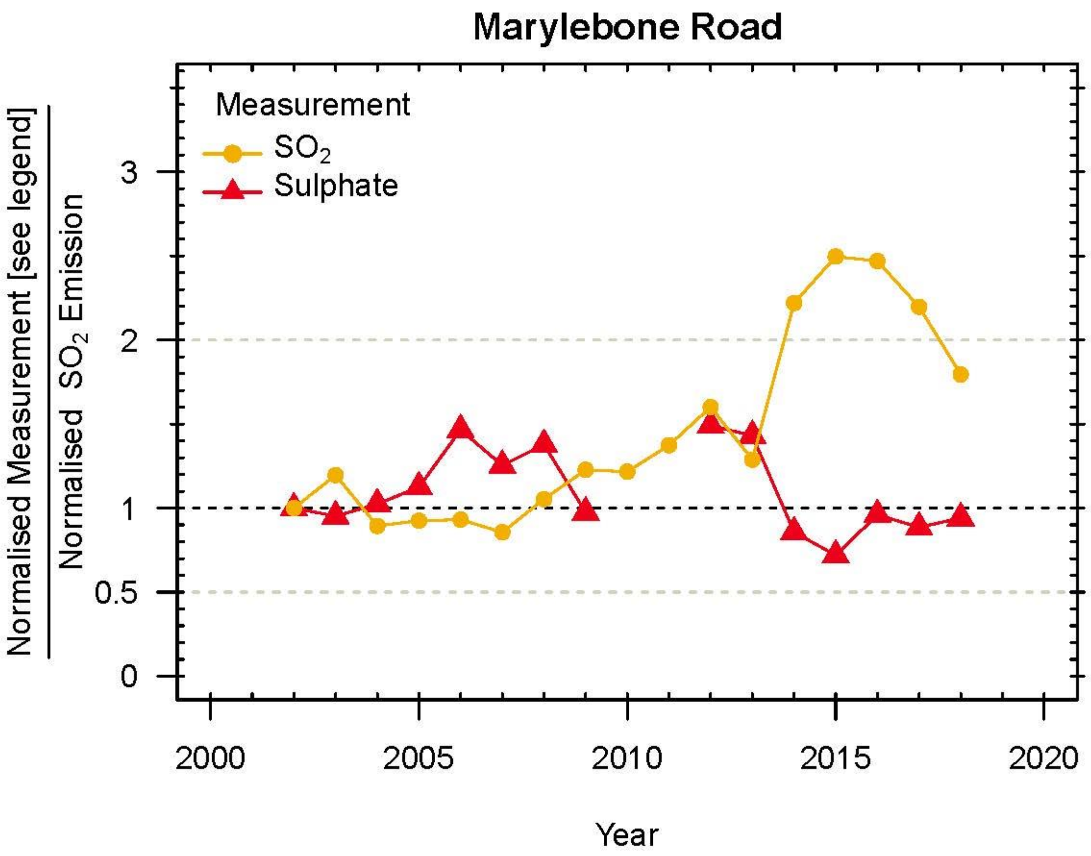

Supplementary Figure 12. Normalised ratios of concentrations of sulphate and SO_2 to emissions of SO_2 at Marylebone Road, 2002 – 2018.

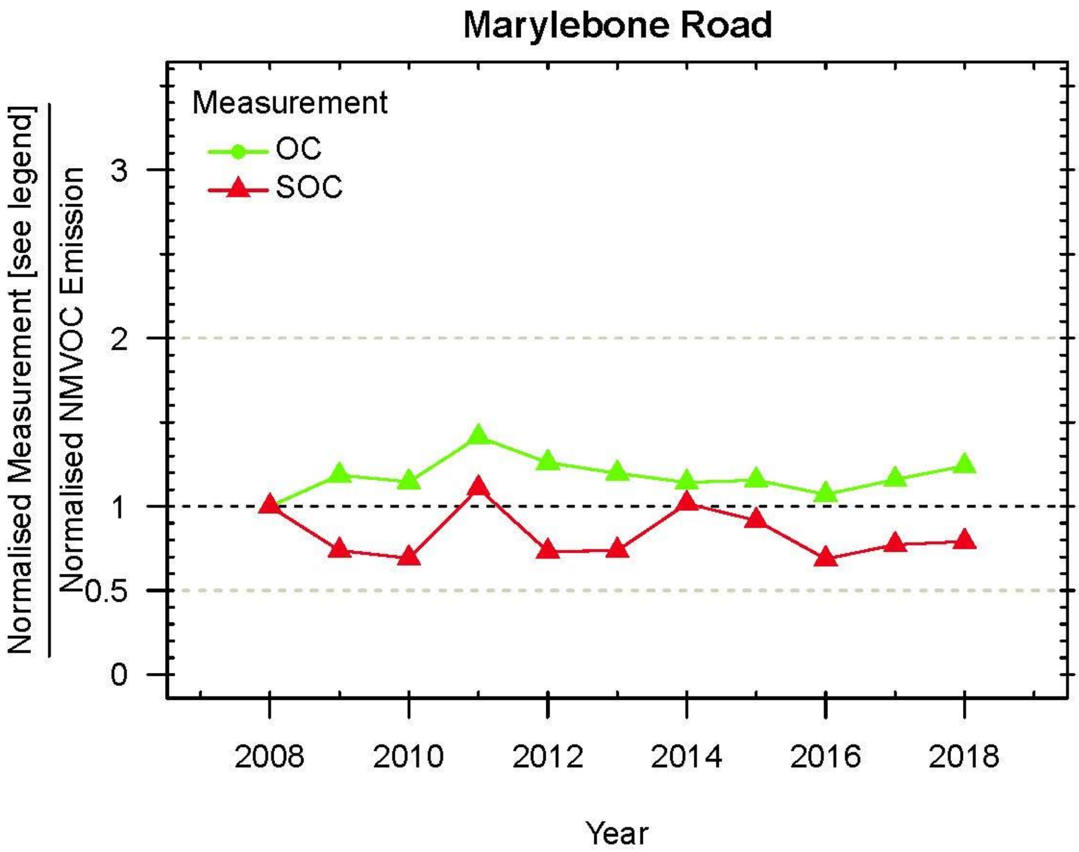

Supplementary Figure 13. Normalised ratios of concentrations of SOC and OC to emissions of NMVOC at Marylebone Road, 2002 –2018.

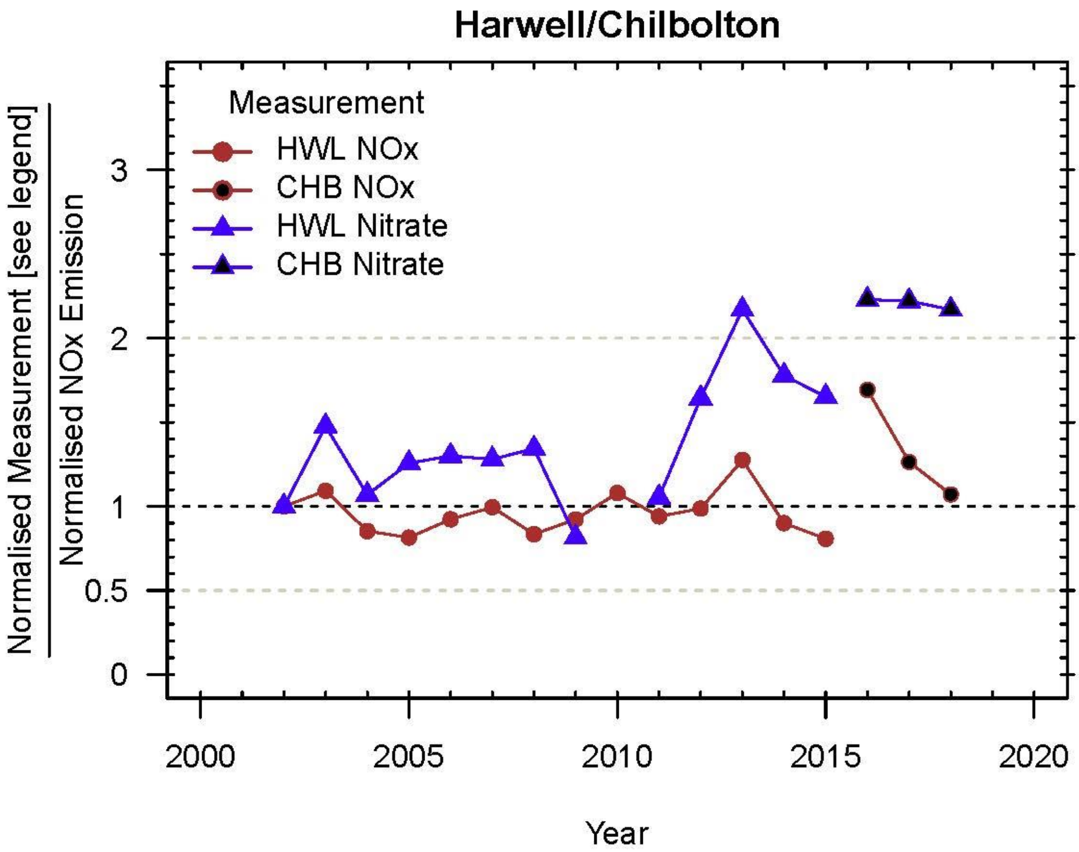

Supplementary Figure 14. Normalised ratios of concentrations of nitrate and NO_x to emissions of NO_x at Harwell and Chilbolton, 2002 – 2018.

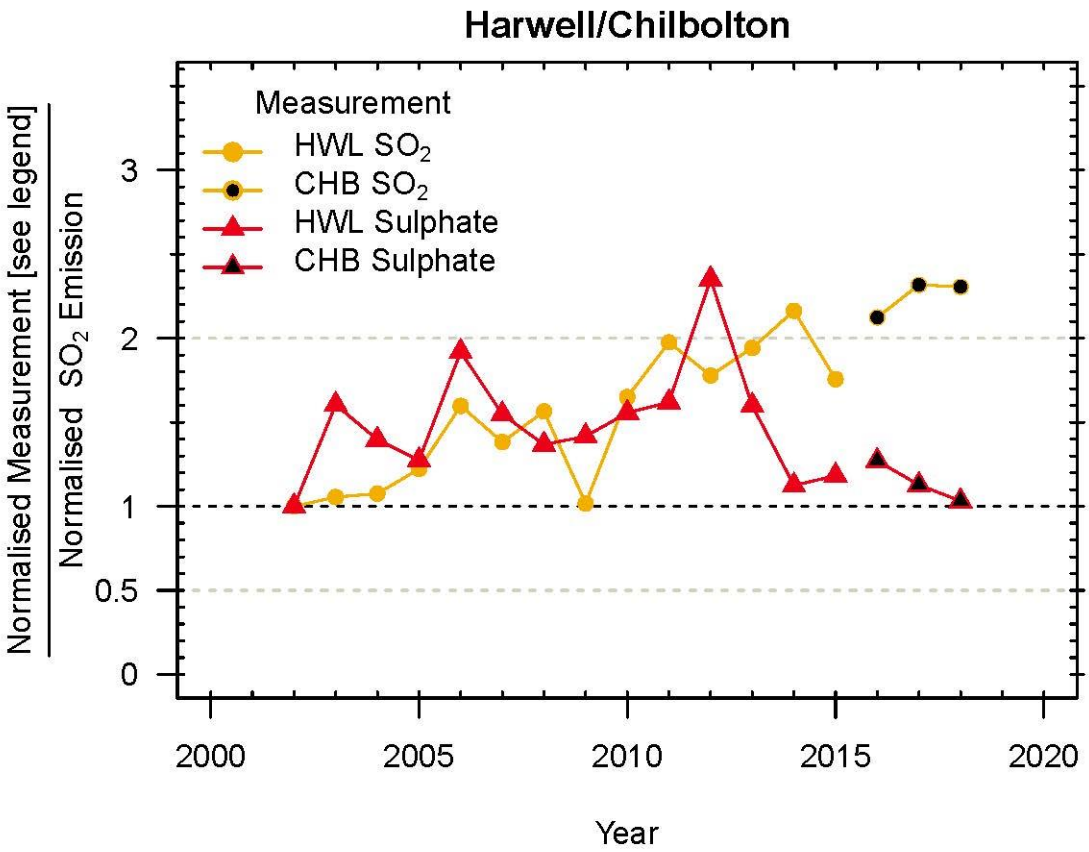

Supplementary Figure 15. Normalised ratios of concentrations of sulphate and SO_2 to emissions of SO_2 at Harwell and Chilbolton, 2002 – 2018.

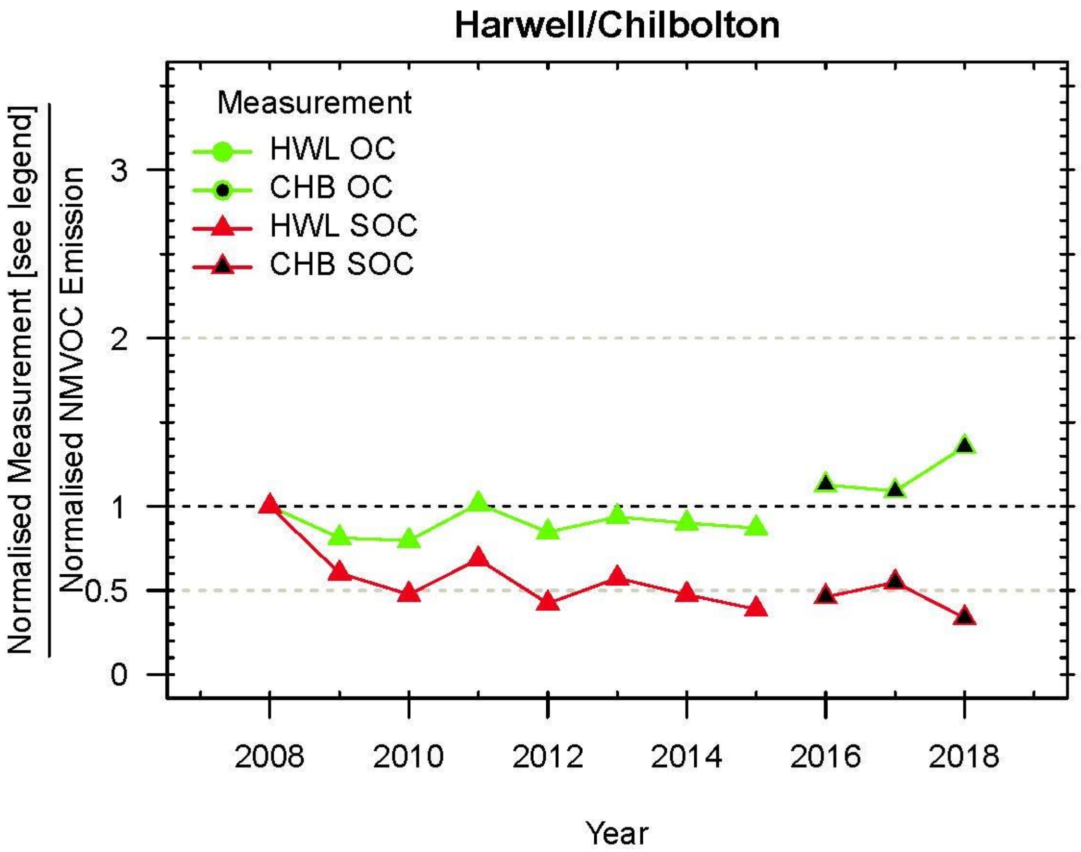

Supplementary Figure 16. Normalised ratios of concentrations of SOC and OC to emissions of NMVOC at Harwell and Chilbolton, 2008 – 2018.
